# Rock deformation *vs.* radon emission: some constraints from shear stress-controlled experiments

**DOI:** 10.1038/s41598-023-43374-6

**Published:** 2023-09-29

**Authors:** Eleonora Benà, Elena Spagnuolo, Antonio Piersanti, Gianfranco Galli, Claudio Mazzoli, Raffaele Sassi

**Affiliations:** 1 Dipartimento di Geoscienze, Università degli Studi di Padova, Via Gradenigo 6, 35131 Padova, Italy; 2 Istituto Nazionale di Geofisica e Vulcanologia (INGV), Via di Vigna Murata 605, 00143 Rome, Italy

**Keywords:** Environmental sciences, Natural hazards, Solid Earth sciences

## Abstract

Numerous field and laboratory studies have been conducted to investigate the relationship between radon variation and seismic events, as well as the complex link between radon emission and rock deformation mechanisms. However, a clear understanding of this correspondence and systematic observations of these phenomena are still lacking, and recent experimental studies have yet to yield conclusive results. In this study, we investigate the possible relationships between radon migration dynamics and rock deformation at the micro-scale through laboratory experiments using the SHIVA apparatus under shear stress-controlled conditions and simultaneous high-resolution radon measurements. We studied the behaviour of three different lithologies to show that radon emission varies in response to rock deformation and this variation is highly dependent on the mineralogy and microstructure. This study represents the first attempt to define radon gas as an indicator of transient and rapid rock deformation at the micro-scale.

Radon (^222^Rn) is one of the most studied radioelements due to its harmful effect on human health and its significance in understanding the migration through more permeable pathways, such as faults and fractures, within the Earth’s crust and toward the Earth surface. Despite its short half-life (i.e., 3.85 days) Rn can migrate long distances along faults and it is detectable even in very low concentration in the soil gas.

Over the years, numerous field studies have been conducted on Rn behaviour and variation in response to seismic events to evaluate possible relationships. There are many examples of these kind of applications in the literature, including fault localization from Rn concentration enhancement^1^; the use of spatial Rn concentration and numerical simulation of Rn transport to delineate fault geometry^2^; change in Rn concentration in soil gas and dissolved in groundwater before, during, and after earthquakes^3,4^; and the correlation between Rn concentration of soil gas at an active fault, which is sensitive to cumulative recent seismicity^5^. However, unambiguous and systematic data/observations of a possible (causal) relationship between Rn and those phenomena are still lacking.

To this end, laboratory experiments play a key role in understanding the complex relationship between Rn geochemical behaviour and rock deformation mechanisms, as experiments offer a unique opportunity for direct access to the source of Rn. Recent experimental studies include experiments run to test the role of temperature, compression and fracture^6–12^, but are still not conclusive on the role of the applied deformation at close to natural seismic cycle deformation conditions.

In this research, we investigate the possible relationships between radon migration dynamics and rock deformation through laboratory experiments using the rotary shear apparatus SHIVA (Slow to HIgh Velocity Apparatus, see Di Toro et al.^13^) under shear stress-controlled conditions (“torque tests”, see Cornelio et al.^14^ for details) on a pre-existing fracture in frictional contact under a constant normal stress and simultaneous continuous radon measurements using forced air circulation in a closed system from the sample holder to the radon-detector, in the absence of any other type of fluid transport. We studied the behaviour of three lithologies (paragneiss, granite and orthogneiss) characterised by different mineralogic composition and microstructure. The rock types were sampled from the crystalline basement of the Pusteria Valley (north-eastern Alps, Bolzano, Italy), a well-known area from a geological and structural point of view. In particular, the lithologies belong to the main outcrops along the Pusteria fault system characterised by a wide fractured zone and a high gas permeability^15^.

This work combines: (1) observations derived from rock deformation tests and continuous radon monitoring; (2) high resolution (i.e. high sampling frequency) Rn time series analysis through robust statistical approach; (3) petrographic analysis of mineral phases and microstructure by optical microscope. This study represents the first attempt to relate variations in radon gas (measured at high frequency—approximately 1 Hz) through alpha decays in a closed loop system, lithology (by testing three different mineral assemblages from the same natural case study) and rock deformation (under seismic deformation condition at shallow depth) at the micro-scale.

## Results

### Petrographic analysis

**Figure 1:**
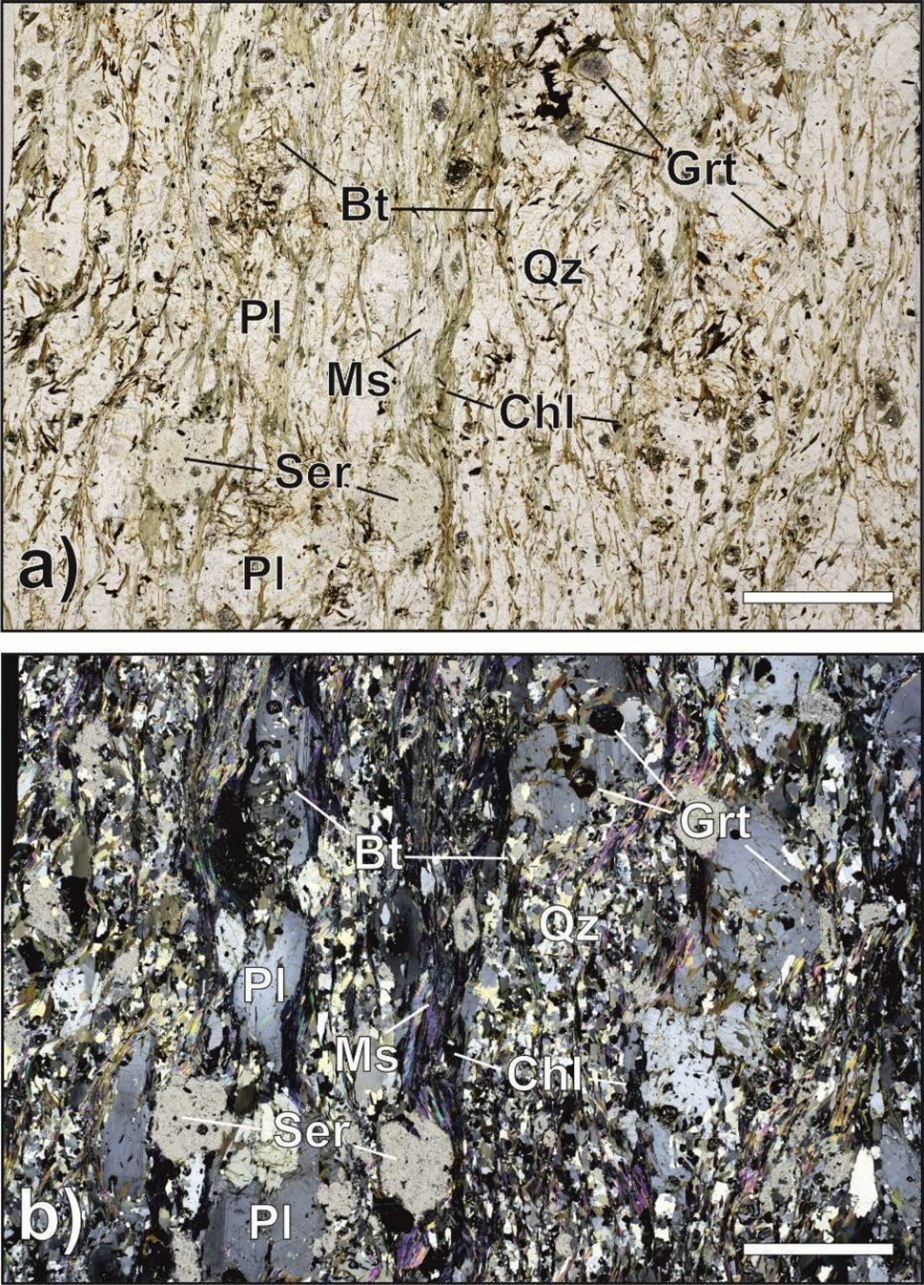
(**a**, **b**) Paragneiss. Plane- (**a**) and cross-polarised light (**b**) photomicrographs of paragneiss. Schistosity is planar to gently undulated and it is defined by the orientation of the sheet silicates (nearly vertical in the picture). The layering is determined by muscovite (Ms)-, biotite (Bt)-, sericite (Ser)-, chlorite (Chl)-rich bands alternating with granoblast-rich layers mainly composed of quartz (Qz) and plagioclase (Pl). Scale bar for references measures 500 µm. Mineral abbreviations after Warr^18^.

**Figure 2:**
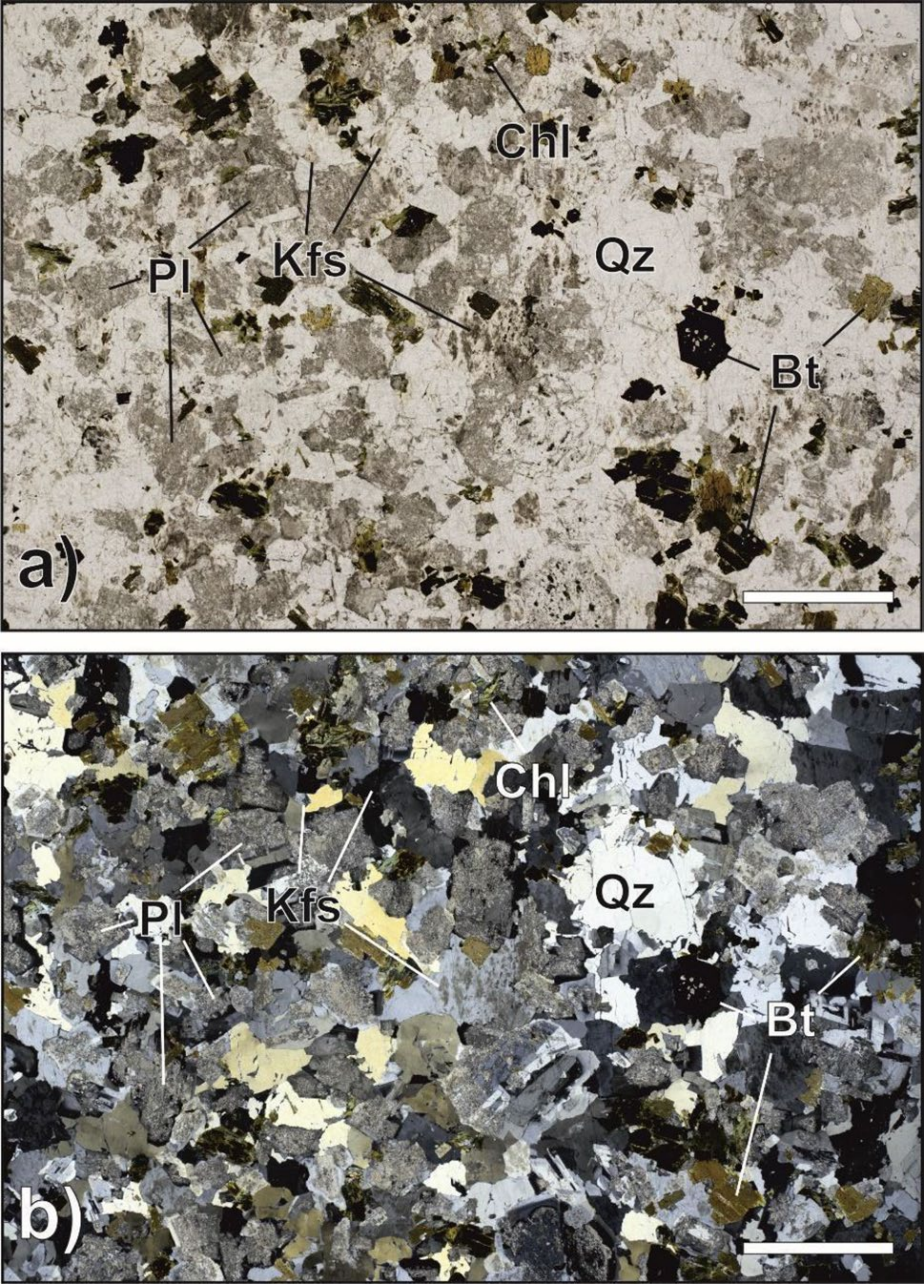
(**a**, **b**) Granite. Plane- (**a**) and cross-polarised light (**b**) photomicrographs of granite. The granite has a heterogranular isotropic structure and is characterised by a mineral assemblage consisting of potassium feldspar (Kf), plagioclase (Pl), quartz (Qtz), and biotite (Bt). Hydrothermal activity has strongly altered the primary mineral phases, such that Bt has been extensively replaced by chlorite (Chl), Pl by a mesh of muscovite, epidote, and calcite (greyish coloured dusty pattern in Fig. 2a). Scale bar for references measures 500 µm. Mineral abbreviations after Warr^18^.

**Figure 3:**
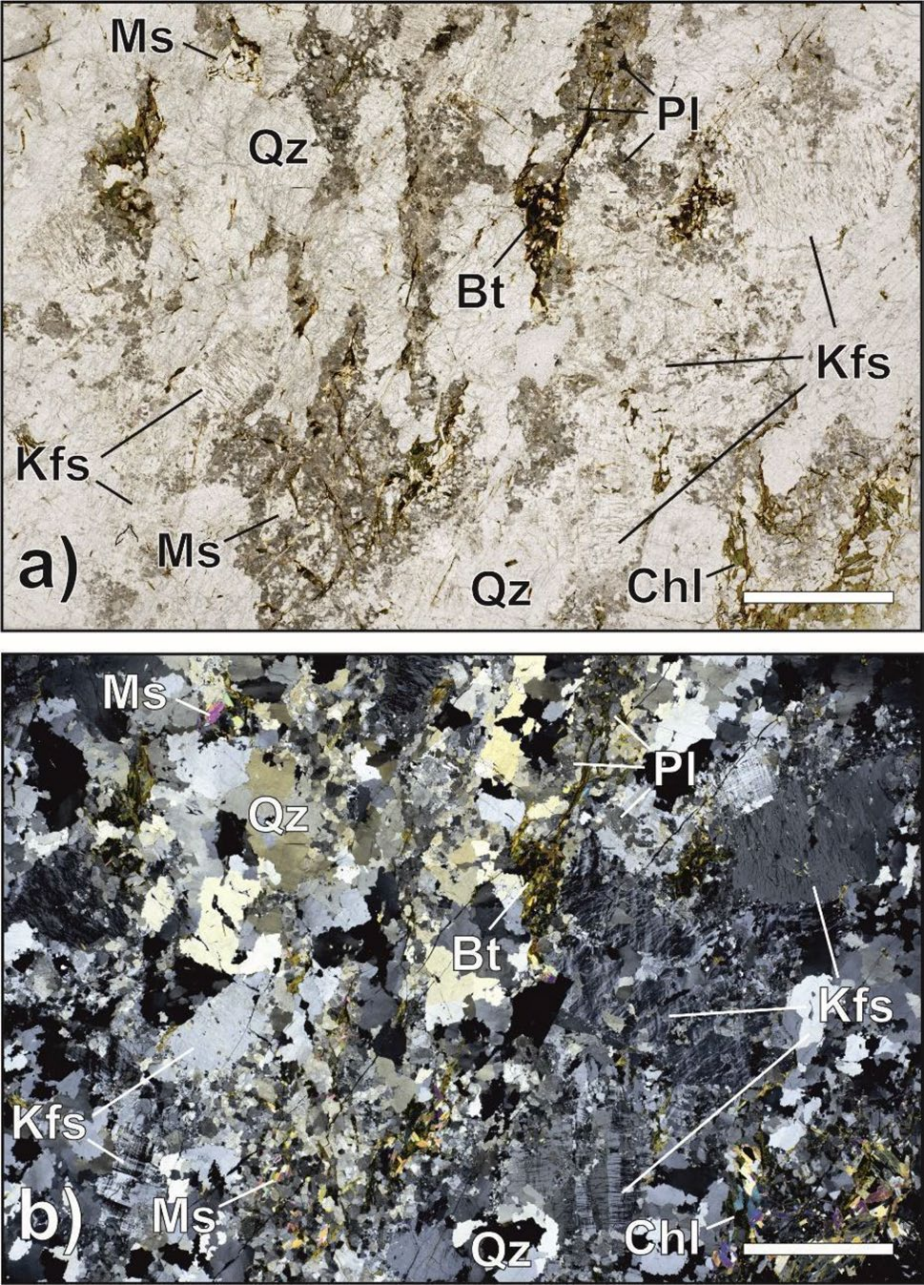
(**a**, **b**) Orthogneiss. Plane- (**a**) and cross-polarised light (**b**) photomicrographs of orthogneiss. Gneissic structure is determined by the orientation of large porphyroclasts of Kf (up to 7 mm), discontinuous bands of sericitized plagioclase crystals (greyish coloured dusty pattern in Fig. 5a), and phyllosilicates (mainly Chl and Ms). Biotite in these rocks has undergone significant alteration into Chl. Scale bar for references measures 500 µm. Mineral abbreviations after Warr^18^.

We conducted petrographic analysis of the non-deformed (pre-experiment) lithology of paragneiss (Fig. 1a,b), granite (Fig. 2a,b) and orthogneiss (Fig. 3a,b).

### Paragneiss

Samples s1896 and s1904 (Fig. 1a,b) belong to the Austroalpine basement outcropping to the south of the DAV line near the municipality of Falzes. Schultz^16^ and Mazzoli et al., (2002)^17^ estimated a Variscan metamorphism of 0.7–0.8 GPa and 630 °C in this area. The samples are fine- to medium-grained, light-brown to grey paragneiss, characterised by a mineral assemblage consisting of biotite (Bt), muscovite (Ms), chlorite (Chl), garnet (Grt), plagioclase (Pl) and quartz (Qtz) (mineral abbreviations after Warr^18^). They exhibit a schistose, porphyroblastic, and layered structure. Schistosity is planar to gently undulated and is defined by the orientation of the sheet silicates. The porphyroblastic structure is related to the presence of Grt and Pl porphyroblasts. The layering is determined by millimetric sheet silicates-rich bands (Ms, Bt, Chl) alternating with granoblast-rich layers mainly composed of Qtz and Pl. The grain size of sheet silicates in mica-rich bands is mainly around 0.5–1 mm whereas the dimensions of Qtz and Pl grains generally fall in the range 0.5–1 mm. Plagioclase may form 3–4 mm porphyroblasts. Millimetric pseudomorphic aggregates of sericite and subordinate chlorite after staurolite are also present. Accessory phases such as tourmaline, apatite, opaque minerals, zircon, and epidote are also present.

### Granite

Samples s1895 and s1897 (Fig. 2a,b) are representative of the Southalpine Permian granite^19^ near the Periadriatic Lineament in the municipality of Mules. The granite has a heterogranular seriate isotropic structure and is characterised by a mineral assemblage consisting of potassium feldspar (Kfs), plagioclase (Pl), quartz (Qtz), and biotite (Bt) (mineral abbreviations after Warr^18^). The Kfs and Pl crystals are subhedral and euhedral, respectively. The grain size distribution of quartz, plagioclase and K-feldspar ranges mainly between 0.2 and 5 mm. Hydrothermal activity has strongly altered the primary mineral phases, such that Bt has been extensively replaced by chlorite (Chl), Pl by a mesh of muscovite, epidote, and calcite, and Kfs has been altered to varying degrees into kaolinite. Accessory phases such as epidote, apatite, zircon, and opaque minerals are also present.

### Orthogneiss

Samples s1916 and s1917 (Fig. 3a,b) are representative of the Austroalpine basement. Granitic orthogneisses are light-grey rocks that resemble granite, composed of minerals such as potassium-feldspar (Kfs), quartz (Qtz), plagioclase (Pl), chlorite (Chl), muscovite (Ms), and biotite (Bt) (Mineral abbreviations after Warr^18^). These rocks formed through regional metamorphism of a granitic body in the late Ordovician period^20^.

The degree of foliation in these rocks can range from barely perceptible to gneissic, and it is determined by the orientation of large porphyroclasts of Kfs (up to 7 mm), discontinuous bands of sericitized plagioclase crystals, and phyllosilicates (mainly Chl and Ms). Biotite in these rocks has undergone significant alteration into Chl. Additionally, decussate Ms flakes (0.1 mm) often crystallise on chloritized biotite layers. The grain size of these rocks varies, with Kfs and Qtz exhibiting coarse grains (7 mm) and Pl exhibiting medium grains (1 mm). Very small, rare garnet grains can also be found within Pl crystals. Accessory minerals such as apatite, epidote, zircon, opaque minerals are also present in these rocks.

### Shear stress-controlled experiments

**Figure 4:**
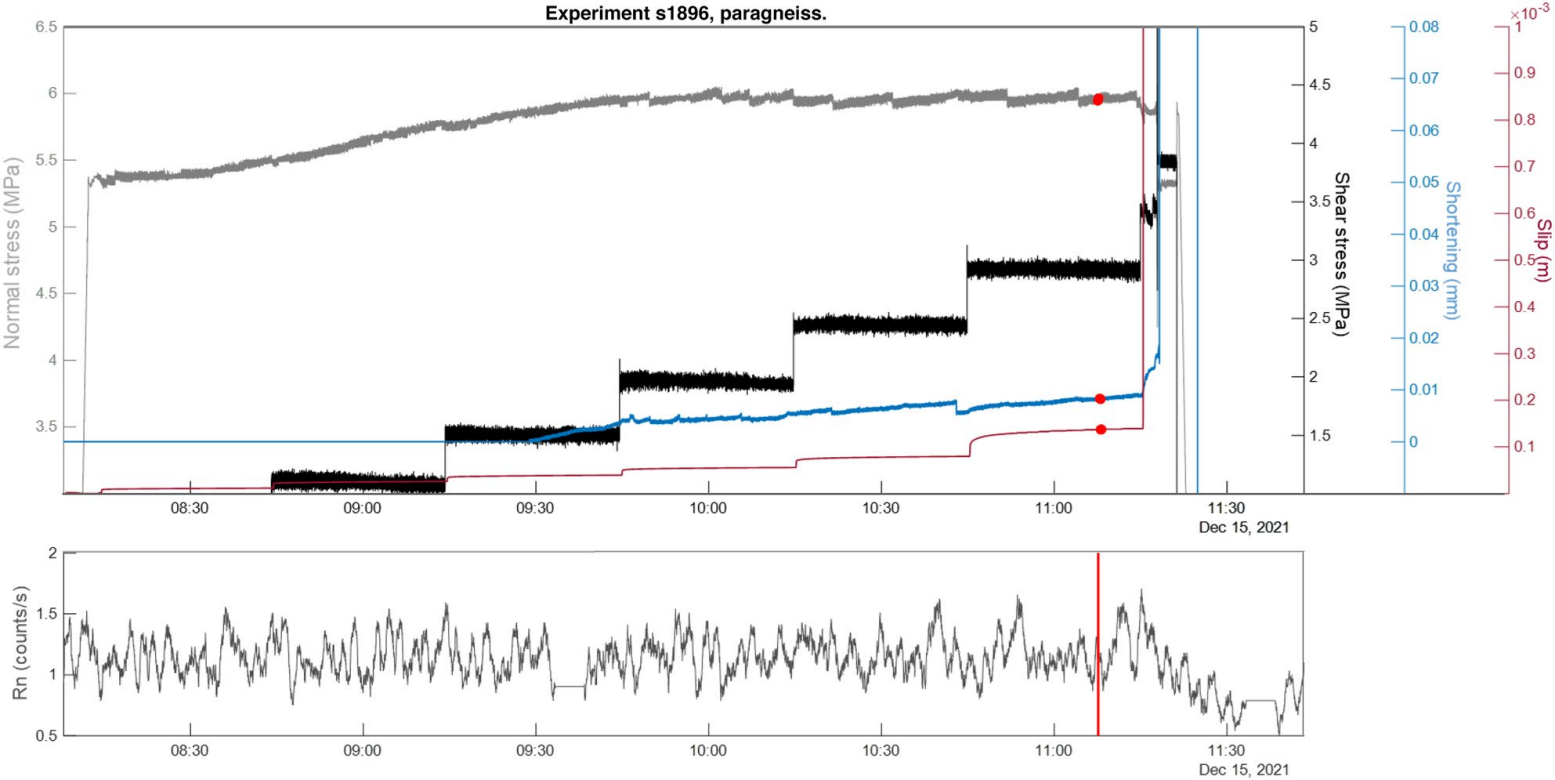
Top panel: Normal stress (MPa, dark grey solid line), shear stress (MPa, black solid), slip (m, red solid), and axial shortening (mm, blue solid); change points (algorithm “std”, red circle). Bottom panel: radon, count/s moving average over 1 min, red lines change points (algorithm “std”).

**Figure 5:**
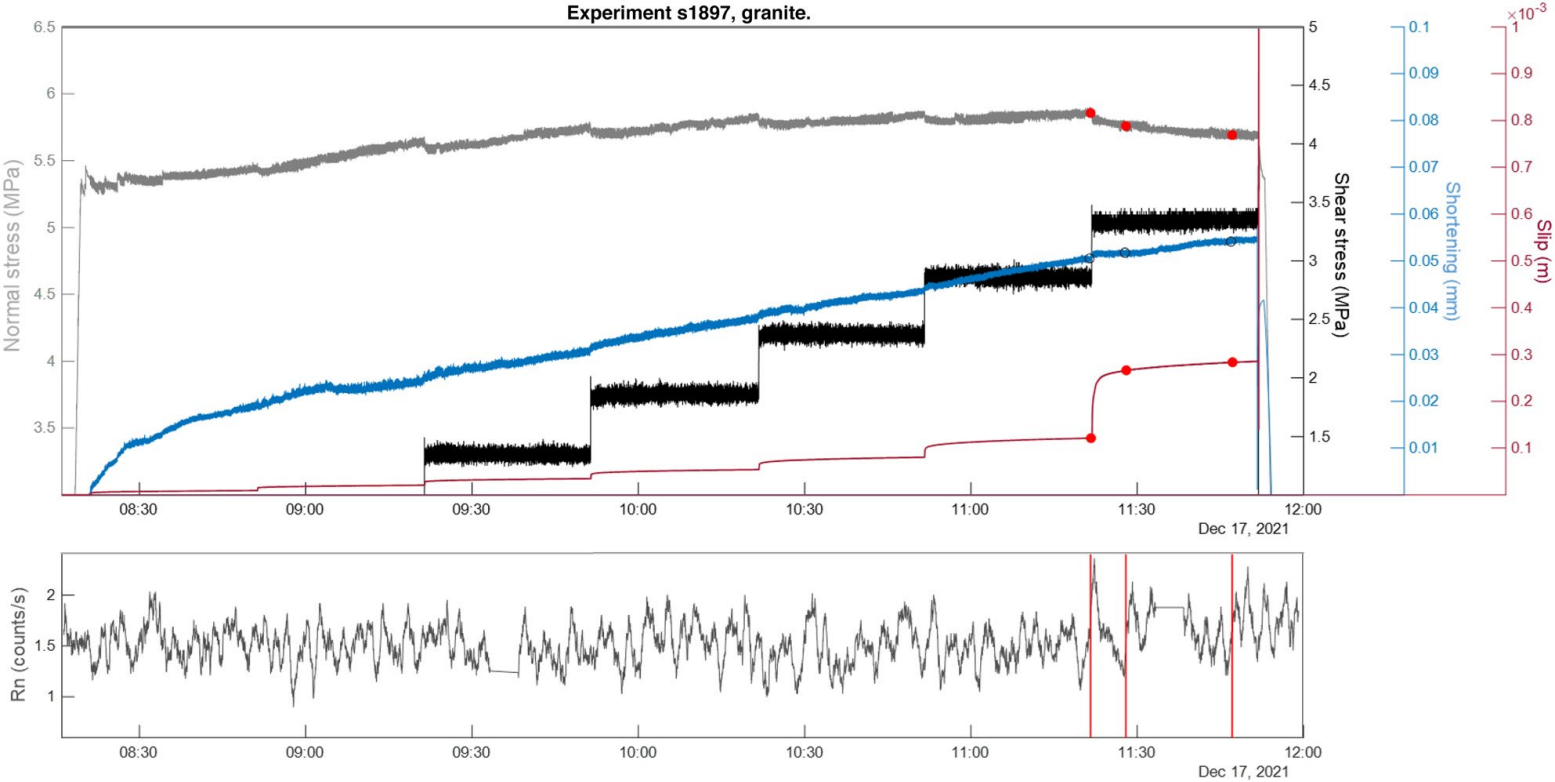
Top panel: Normal stress (MPa, dark solid line), shear stress (MPa, black solid), slip (m, red solid), and axial shortening (mm, blue); change points (algorithm “mean”, red circles). Bottom panel: radon, count/s moving average over 1 min, red lines change points (algorithm “mean”).

**Figure 6:**
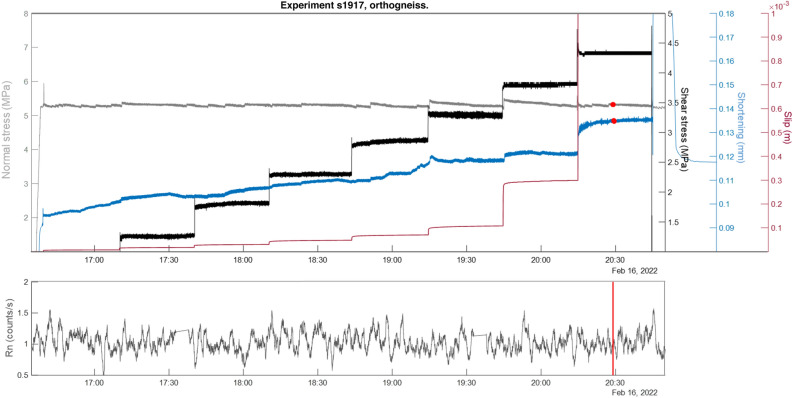
Top panel: Normal stress (MPa, dark solid line), black shear stress (MPa, black solid), slip (m, red solid), and axial shortening (mm, blue solid); change points (algorithm “mean”, red circles). Bottom panel: radon, count/s moving average over 1 min, red lines change points (algorithm “mean”).

We conducted six shear stress-controlled tests (two tests per lithology). During the experiment the shear stress was increased stepwise (black solid line in Figs. 4, 5 and 6), while the slip (red solid line) and the slip-velocity adjusted spontaneously. Each stepping caused variations in the axial shortening (blue solid line) and normal stress (light grey solid line). The Rn counts recorded in continuous during the stepping stage are shown on bottom panels of Figs. 4, 5 and 6 in grey colour for the paragneiss (s1896), granite (s1897) and orthogneiss (s1917) respectively.

### Paragneiss

In the case of experiment s1896 (Fig. 4) Rn counts exhibit a constant trend throughout the experiment with a decrease near the main instability which occurred at a shear stress of 3 MPa (friction coefficient = 0.6).

For this experiment we present the change-point analysis performed on the Rn time series using the "std" algorithm, which identified two overlaid change points (i.e. 15 s apart). Remarkably, the two change points were detected close to the main instability. The main instability was anticipated by a slight increase and accompanied by a sharp decrease in Rn counts although by visual inspection a possible link with the mechanical data was unclear. This link was rather established using the change point analyses which identified two points marking region of nearly stable behaviour of all the measured variables nearly 11:07 min before all of them started to transiently change: normal stress decreased of 0.2 MPa, slip accelerated towards the main instability and axial shortening reported a significant change in the compaction rate (nearly 0.01 mm/min) before the sharp increase recorded during the main instability.

Experiment s1904 (see supplementary materials, Fig. S1) also exhibited a nearly constant trend in Rn counts throughout the experiments with a sharp decrease in Rn counts close to the main instability which occurred at a shear stress of 3.3 MPa (friction coefficient = 0.6).

For this experiment we present the change-point analysis performed on the Rn time series using the "lin" algorithm, which identified three change points. The first two change points were located close to an increase in Rn counts compared to the average Rn trend. In correspondence to these two change points, the mechanical data showed a progressive increase in the normal stress and axial shortening with no significant changes in either shear stress or slip. The third detected change point instead identified a sharp decrease in Rn counts about 3 min after the main instability. The significance of this third point though shall be analysed considering that the occurrence of the main instability in this experiment was forced by a sign error in the manual control of the shear stress which resulted in a clockwise rotation of the column. Conversely to all the other experiments, the main instability did not occur spontaneously. Despite this technical issue, again the Rn decrease at change point location also corresponded to a sharp increase in slip and compaction of the rock samples.

### Granite

Experiment s1897 (Fig. 5) shows a constant trend in Rn counts throughout the duration of the experiment and a slight increase in Rn count close to the occurrence of the main instability at a shear stress of 3.4 MPa (friction coefficient = 0.7).

For this experiment we present the change-point analysis performed on the Rn time series using the "lin" algorithm, which identified three change points.

All three change points were located near the rock instability, close to the first increase in Rn counts. Specifically, the three change points marked three stages of the last shear stress step (during the stepping up and before the main instability event) where the normal stress decreased of 0.2 MPa and slip increased up to 0.3 m. Axial shortening gradually increased throughout the experiment suggesting constant compaction up to 0.06 mm at the end of the experiment.

Experiment s1895 in granite (see supplementary materials, Fig. S2) shows a constant trend in Rn counts during the first two hours of the experiment (8.30 to 11:00 a.m.) and a gradual increase throughout the duration of the experiment until the onset of the main instability which occurred at shear stress of 4 MPa (friction coefficient = 0.8), slightly higher than the granite sample s1897. After the instability, Rn counts returned to a constant trend.

For this experiment we show the change-point analysis performed in the Rn time series using the "mean" algorithm, which identified three change points. The first change point was located near the first increase in Rn counts trend, coeval to a gradual increase in the axial shortening due to a gradual compaction of the samples. The last two change points were located a few minutes apart in correspondence to another gradual increase in Rn counts (maximum value 3.6 counts/s) about 25 min before from the main instability occurring at time 12:12 which caused a fast and self-arresting event (slipping at nearly 0.2 m/s). The trend shown in the mechanical data is consistent with the detected change points, as they correspond to a sharp increase in slip and increase in the shortening (up to 0.06 mm). This experiment was manually stopped after the occurrence of this small instability which resulted in a thin layer of pseudotachylites visible over the contact surface of the recovered samples suggesting that the experiment experienced a large temperature increase. The manual stop was forced by an overheating error of the control system.

### Orthogneiss

In the case of experiment s1917 (Fig. 6) Rn counts exhibited a constant trend throughout the experiment; the main instability occurred at a shear stress of 4.3 MPa (friction coefficient = 0.8).

For this experiment we present the change-point analysis performed in the Rn time series using the "std" algorithm, which identified two overlaid change points (i.e., 9 s apart). These two change points were located at the last stage of the stepping in shear stress, before the main instability and after the occurrence of a short but fast unstable event (time c.a. 20:14) running at 0.14 m/s (see supplementary materials, Fig. S4). An initial dilation was recorded right before the occurrence of this fast event which was followed by a sharp increase in axial shortening (= compaction). Although no particular trends are visible in the Rn series, the change points highlighted clear variations in the mechanical data.

In case of experiment s1916 (see supplementary materials, Fig. S3), Rn counts exhibit a constant trend throughout the experiment, except for a sharp increase after the rock instability occurring at a shear stress of 3.5 MPa (friction coefficient = 0.7).

For this experiment we present the change-point analysis performed in the Rn time series using the "mean" algorithm, which detected two change points. It is worth noting that this experiment is the only one in which the first identified change point was located at the beginning of the experiment. This change point marked a slight increase in Rn counts, which is related to the initial rise in normal stress, and shortening can be observed, linked to the first sample compaction. The second change point was located about 2 min after an unstable event running at slip velocity of 0.1 m/s. This event was preceded by a short dilation (time 20:30) before the sample started compacting and normal stress slightly decreased. At the end of this event and until the end of the experiment we detected a slight increase in Rn counts.

## Discussion

The experiments conducted on three different lithologies revealed two important features: (i) the three lithologies exhibited distinct trends in radon emissions and (ii) the change-point analysis performed on the Rn time series effectively marked significant stages of the experiment and confirmed a casual variation between radon variations and transient stages of the mechanical data. The strength of this statistical approach lies in the remarkable consistency of results obtained from all the algorithms used. These algorithms not only identified the most significant variations in radon counts, but also highlighted specific mechanical features as all the change points coincided with relevant variations in mechanical behaviour. Notably, the results obtained from all the adopted algorithms consistently indicated a strong correlation with the mechanical data. Additionally, due to the nature of this technique, it is necessary to set a maximum number of allowed change points. In this study, the maximum number of points was not always assigned, and when assigned, they often appear at the same point (e.g. experiments s1896 and s1917). This serves as a strong indication of the high significance of the performed statistical analysis.

The two paragneisses (i.e. s1896, s1904) exhibited a fairly constant radon trend throughout the experiment, with slight decrease at the onset of the main instability event. The observed radon trend in paragneiss can be attributed to the specific mineralogy and microstructure of this rock. Paragneiss is a relatively soft rock due to the presence of millimetric iso-oriented layers made mostly of sheet silicates (45% of the volume). Under the applied stress, these sheet silicate flakes are further oriented and compacted, forming an almost impermeable barrier preventing radon exhalation from the sample surface. This phenomenon may explain the decrease in radon counts close to and during the main instability. The change-point analysis highlighted that variation in radon counts are consistent with the mechanical data, where the two overlaid change points in the case of e.g. experiment s1896, were detected at the beginning of radon decrease close to the main instability.

On the other hand, granite behaves differently. Both experiments s1895 and s1897, although run at slightly different stress conditions due to a technical challenge, showed a gradual increase in radon counts, reaching the maximum values close to and during the main instability. This slight increase in Rn counts corresponded to a gradual increase in axial shortening throughout the experiment. The change-point analysis confirmed that radon variations are reflected in the mechanical data, where three change points were detected in both granites at the beginning of radon increase and before the main instability. The mineralogical and microstructural features of the granite produce a different radon trend compared to paragneiss. Granite is a magmatic rock mainly composed of granular minerals such as quartz and feldspars, with only small amounts of sheet silicates (8% of the volume), this makes this lithology hard and compact. Under the load application (both normal and shear stress), granular minerals such as quartz and feldspars are fractured, gradually decreasing their average grain size and increasing the microscopic porosity, increasing the sample gas permeability^21^. Therefore, it is likely that during gradual sample fracturing, a greater fraction of radon escapes from the sample surface.

In the case of the two samples of orthogneiss (i.e. s1916, s1917) we documented a slight increase in radon counts after the main instability in the first experiment, but no significant trend is visible in the second experiment. Both experiments evidenced the onset of a short but fast (c.a. 0.1 m/s of slip velocity) event before the main instability, anticipated by a short phase of dilatancy. The change-point analyses marked the occurrence of these events in the respective radon time series. Orthogneiss displayed a behaviour that is midway between granite and paragneiss in terms of microstructure. The granoblastic layers made up of granular quartz and feldspars show a decreasing grain size due to increasing fracturing, leading to an increase in microscopic porosity^21^ and conferring a higher gas permeability similar to granite. However, at the same time, the lepidoblastic bands made up of sheet silicates (15% of the volume) are compacted, forming an almost impermeable barrier preventing radon exhalation from the fracture, similar to paragneiss.

Therefore, during the load application (both normal and shear stress) in orthogneiss, the increased Rn counts likely derived from the increased porosity of granular layers (probably leading to dilatant stages) is compensated by a decreased Rn emission due to sheet silicate flakes compaction in lepidoblastic bands, sealing the porosity and preventing gas flow through the rock. This mixed structure made of granular materials and clays exhibited alternating dilatant/compacting behaviour as documented by the recording of the axial shortening which is also significantly different from either granite or paragneiss. In these experiments, our aim was to control and isolate only a few of the many phenomena that can occur at the natural scale and lead to radon variations at the surface. In nature, various factors, including the presence of carrier fluids, can affect the emission, concentration and migration of radon. Additionally, faulting and other geological processes can increase radon emission, but this behaviour is not always consistent: in some cases, a decrease or insignificant variation in the radon trend can also occur. Therefore, it is crucial to carefully analyse the specific petrographical and microstructural features at a micro-scale, as well as the geological and structural conditions of the area at a macro-scale to understand Rn behaviour in a complex tectonic setting. Although external fluid dynamics are absent in these experiments, the shear stress control procedure still represents the gradual increase in the tectonic loading which can be observed in nature during the earthquake cycle. The radon counts observed in these experiments can be considered representative of the mechanical behaviour of faults in the represented lithologies, within a very simplified, closed and controlled environment. The duration of laboratory experiments greatly compresses the timescales of the real seismic cycle, and the temporal resolution of events is proportionally reduced. Nevertheless, in several cases, we have evidence of significant radon changes (i.e., detectable by Change Point Analysis, see section Change Point Analysis in Methods) before the onset of mechanical instabilities.

## Conclusions

The shear stress-controlled experiments, carried out on the three different lithologies (paragneiss, granite, orthogneiss) highlight a significant phenomenon involving a direct and almost instantaneous correlation between rock deformation and radon variation, which is also dependent on lithology. By enhancing temporal resolution of the experiments (close to real-time measurements) clear trends and transient variations in radon emissions by the rock samples can be identified and correlated with specific and transient features in the mechanical data. Rn emission varies according to rock deformation and is highly dependent on lithology, mineralogy and microstructure. This behaviour is evident in all the three considered lithologies, paragneiss displays a sharp decrease in radon counts during the main instability, granite shows a gradual increase in radon counts throughout the experiment, and orthogneiss exhibits a behaviour intermediate between granite and paragneiss, in terms of microstructure, with no significant increase or decrease in radon counts. Robust statistical tool (i.e. change point analysis) is able to identify frictional instabilities from independent Rn measurements, highlighting a clear correlation between Rn emission and the mechanical behaviour of rocks. This relation is measurable in a short time frame (minutes), and it is lithology dependent (e.g. not all rocks are expected to create easy pathways for radon emission during frictional instabilities). These observations are reliable in a simplified, closed and controlled environment but, starting with the micro-scale, help to establish limitations on the utilisation of radon gas as an indicator for transient and rapid rock deformation in complex natural scale phenomena.

## Methods

### Petrographic analysis

Rock samples of paragneiss, granite and orthogneiss were collected from the crystalline basement located in the Pusteria Valley (north-eastern Alps, Bolzano Province, Italy). The collected samples were thin-sectioned to a thickness of 30 µm and analysed using a polarising transmitted-light optical microscope.

### Shear stress-controlled experiments

**Figure 7:**
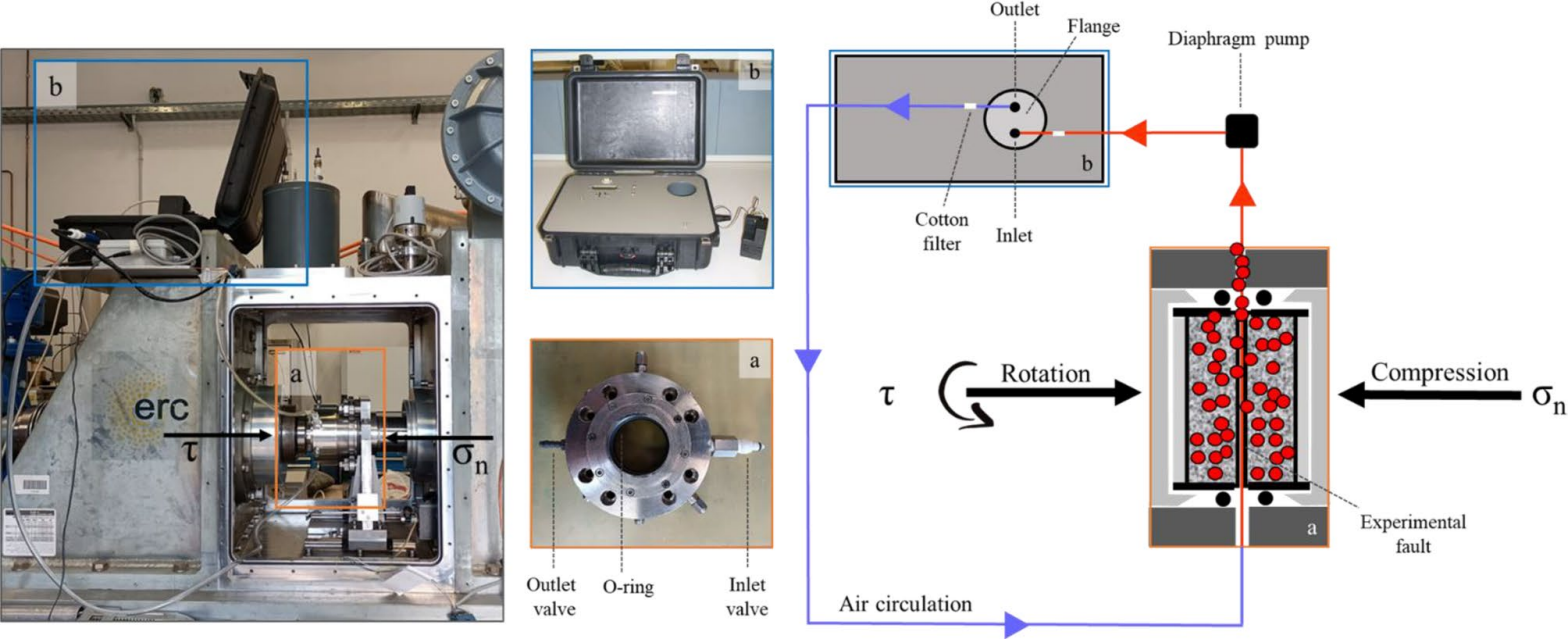
The experimental setup. In the right panel is shown a schematic representation of the experimental setup consisting of (**a**) the pressure-vessel connected with polyethylene tubes with (**b**) the radon detector in a closed loop system. Both were built on the rotary shear apparatus SHIVA made of a rotary axis (τ, rotation) and a stationary axis (σ_n_, compression). The air flux is forced from the pressure-vessel to the radon detector with a diaphragm pump (black square). The blue line represents the air circulation in the closed loop; specifically, the red line represents the sensible air volume < 10 ml. In the central panel is reported a zoom of the (**a**) pressure-vessel provided with two Teflon O-rings to isolate the sample assembly; inlet and outlet valves; (**b**) the radon detector.

The experiments were conducted using two samples of paragneiss, granite and orthogneiss. Each rock sample was drilled into two bare-rock cylinders with an external diameter of 50 mm. The bare-rock cylinders were then fixed into aluminium jackets using an inert glue (H40 Kerakoll). Furthermore, the rock samples within the jacket were rectified using a lathe to ensure parallelism of the contact surfaces^22^ representing the experimental fault under investigation. The rock samples were initially placed in a pressure-vessel^23^, a stainless-steel device built on the rotary shear apparatus SHIVA (Slow to High Velocity Apparatus) located in the High Pressure-High Temperature (HPHT) laboratory at the *Istituto Nazionale di Geofisica e Vulcanologia* (INGV) in Rome (Italy). The experimental setup is shown in Fig. 7. The pressure vessel was equipped with two Teflon O-rings to ensure complete isolation of the rock samples from the external environment. Inlet and outlet valves were connected to the radon detector, creating a closed-loop system that maintained consistent moisture and temperature conditions. Regarding the shear stress-controlled experiments, the SHIVA apparatus is capable of simulating the seismic cycle under conditions that approximate natural seismic deformation at depths typical of the shallow upper crust. Initially, the prepared bare-rock samples were brought into frictional contact under a constant normal stress of 5 MPa. Subsequently, the pressure vessel was assembled on SHIVA and connected to the radon detector in a closed loop (Fig. 7). Two experiments were conducted per lithology on SHIVA to ensure data reproducibility and investigate the radon response to deformation using a shear stress-control protocol^14^. These tests proved particularly useful in studying radon dynamics in a fault system due to sample deformation and evaluating its behaviour near a seismic instability.

The shear stress-controlled experiments (Table 1) involved a step-wise increase in the shear stress (0.5 MPa, in a time interval of 30 min) under a constant normal stress of 5 MPa. The slip and velocity evolved spontaneously to adjust the stress state on the experimental fault. The stress stepping concluded at sample failure, characterised by fault weakening, stress drop and rapid rotation of the rotary column of the SHIVA apparatus. During this step which we referred to as the “main instability”, the velocity increased up to a manually set target velocity of 1 m/s. Prior to the main instability, other types of slip instability such as accelerated creep or fast but self-arresting events, were detected, resulting in slip velocities reaching a few cm/s (reported as Fast events in “Notes” in Table 1). After the experiments, the contact surface of the two samples were heavily damaged, making the sample recovery not possible. The damage of the surface explains the erratic trends observed in axial shortening and the measured normal and shear stress over time.

**Table 1:** The six experiments on the three lithologies and the applied experimental parameters.

Experiment	Lithology	Normal stress (MPa)	Shear stress (MPa)	Notes
s1896	Paragneiss	5	0.5/30 min	
s1904	Paragneiss	5	0.5/30 min	Sign error in the manual step
s1895	Granite	5	0.5/30 min	Fast event; error overheating
s1897	Granite	5	0.5/30 min	
s1916	Orthogneiss	5	0.5/30 min	Fast event, lag in shortening
s1917	Orthogneiss	5	0.5/30 min	Fast event

### Real-time radon time series

Initial Rn counts and variations during the experiments were acquired using an alpha scintillation radon detector (Lucas Cell, Fig. 7b), as implemented by Cannelli et al.^12^ and Galli et al.^24^. Radon entered the detector by diffusion through an inlet filter that traps radon daughters; Rn measurements were performed by counting the decay signals with an acquisition time of 1 s. The radon detector was connected to the pressure-vessel built on SHIVA apparatus using polyethylene tubes, which were fitted with cotton filters at the inlet and outlet valves to prevent particle and dust infiltration. A diaphragm pump with a flow rate of 380 ml/min facilitated air circulation within the closed system, between the inlet valve of the vessel and the outlet flange of the radon detector ensuring the sensible air volume less than 10 ml. This configuration allows air circulation within the vessel and from the vessel to the Rn detector in a closed system. Rn is measured in counts/s to preserve the full characteristics of the time series. Variations in radon counts occurring during the six experiments were evaluated with respect to an initial condition set at the achievement of the secular equilibrium between ^222^Rn and its short-lived progeny which is typically achieved after 3 h. In this pre-experiment phase, the rock samples were pre-stressed under 2 MPa for 36 h (see supplementary materials Fig. S5, radon counts in the pre-experiment phase).

The left panel shows the real configuration of the experimental setup with a) the pressure-vessel and the b) radon detector, in a closed system, built on SHIVA apparatus made of the rotary and stationary axis.

### Change-point analysis

A Bayesian Change-Point (BCP) analysis was performed to quantitatively detect any anomalous variation in the radon time series. The BCP approach was initially developed for studying the Earth’s climate system^25^. A change-point in a time series refers to a moment when a specific statistical property of the signal varies abruptly. Algorithms designed for change-point detection work by minimising specific functions of the original time series subsets, typically involving their mean and deviation. The fundamental operation of a Bayesian Change Point (BCP) algorithm can be summarised as follows:

1. The original time series is divided in two subsections;
2. An estimate of the desired statistical property for each subsection is computed;
3. For each sampling time of the timeseries the deviation of the actual statistical property from its empirical estimate is evaluated and cumulated;
4. The cumulated deviation is minimised by varying the division point (i.e., time instant) between the two subsections: the minimising point is the change point.

When the desired statistical property is the raw mean, the procedure can be visualised in a straightforward manner. Given a time series *r*_*1*_, *r*_*2*_,…*r*_*m*_, *r*_*n*_ being *m* the division time instant we can write mean (Eq. 1) and variance (Eq. 2) of the subsets as follow:

1 $$mean \left( {r_{m} , \ldots r_{n} } \right) = \frac{1}{n - m + 1}\mathop \sum \limits_{t = m}^{n} r_{t}$$

2 $$var \left( {r_{m} , \ldots r_{n} } \right) = \frac{1}{n - m + 1}\mathop \sum \limits_{t = m}^{n} (r_{t} { } - {\text{ mean}}\left( {r_{m} ,...{ }r_{n} } \right))^{2}$$

the change point is the point k corresponding to r_k_ minimizing the total residual deviation D (Eq. 3):

3 $$D = \left( {k - 1} \right)var \left( {r_{1} , \ldots r_{k - 1} } \right) + \left( {n - k + 1} \right)var \left( {r_{k} , \ldots r_{n} } \right)$$

The schematic procedure outlined above can be generalised (and rather complexified) to incorporate statistical properties other than the raw mean and include the possibility of more than one change point. In fact, when more than one change point is allowed, a simple iteration of the above procedure invariably leads to data overfitting and a so-called penalising procedure must be introduced (refer to Lavielle, 2005^26^ and Killick et al., 2012^27^ for technical details). Indeed, BCP algorithms have become a standard statistical investigation tool and several major time series analysis and signal processing software incorporate them granting also easy reproducibility and cross-validation of results. For the present analysis we have used the MATLAB Version R2020b^28^. MATLAB incorporates four versions of the algorithm characterised by different desired statistical properties to minimise in finding the change point as outlined above. The four versions are named mean, std, lin, rms (function “findchangepts”, please refer to MATLAB documentation for the technical details of each version). We adopted all four versions of the algorithms in our calculations.

To begin with, our raw radon time series was subjected to a low-pass filter using a running average at six different intervals of 1, 5, 10, 15, 30 and 60 min (see the six different sampling rates in supplementary materials Fig. S6, example from the experiment s1895).

In the context of this study, the raw data (1 min moving average) has been presented with the specific aim of minimising errors associated with individual measurements. The performed analysis pertains to signal processing and time series analysis, focussing on the investigation of time series and signal processing. In such cases, the signal of interest is often obscured by noise, which can be comparable to or even greater in magnitude than the signal itself. The algorithms employed in this framework are specifically designed to operate effectively in such noisy conditions. In this context, the statistical Poissonian counting error is merely one of several potential sources of noise.

The Matlab BCP algorithm identifies an arbitrary number of change points in the radon time series, with the maximum number of change points being an input parameter of the algorithm. For our study, kmax was set to 3. In the main text, only one of the four tested algorithms are reported for each lithology (e.g. the one that exhibits two change points located closely in time). However, it is important to note that all four algorithms performed similarly with minor differences (refer to supplementary materials, Figs. S7–S12, for complete data). Finally, we compared the results of the BCP analysis with the recorded mechanical data^29^ to verify the correlation between radon variations detected by the change point analysis and transient variations observed in the mechanical data.

### Supplementary Information

Supplementary Information.

## Data Availability

All data generated or analysed in this study are included in the supplementary materials, available at https://doi.org/10.5281/zenodo.7900651 and can be downloaded upon request. We use MATLAB Signal Processing Toolbox v 8.5 (2020b), for change point analysis the documentation is available at https://it.mathworks.com/help/signal/ref/findchangepts.html.
