# Supplementary material for: Rock deformation vs. radon emission: some constraints from shear stress-controlled experiments
Source: Sci Rep. 2023 Sep 29;13:16399. doi: 10.1038/s41598-023-43374-6 (PMC10541892; doi:10.1038/s41598-023-43374-6)
Supplement: Supplementary file 1 — Supplementary Information. [file 41598_2023_43374_MOESM1_ESM.docx]

**Rock deformation *vs.* radon emission: some constraints from shear stress-controlled experiments**

**Authors and affiliations:**

Eleonora Benà^1*^, Elena Spagnuolo^2^, Antonio Piersanti^2^, Gianfranco Galli^2^, Claudio Mazzoli^1^, Raffaele Sassi^1^

^1^Dipartimento di Geoscienze, Università degli studi di Padova, Via Gradenigo 6, 35131 Padova, Italy. eleonora.bena@phd.unipd.it

^2^Istituto Nazionale di Geofisica e Vulcanologia (INGV), Via di Vigna Murata 605, 00143 Rome, Italy

*corresponding author: eleonora.bena@phd.unipd.it

**Figure S1:**


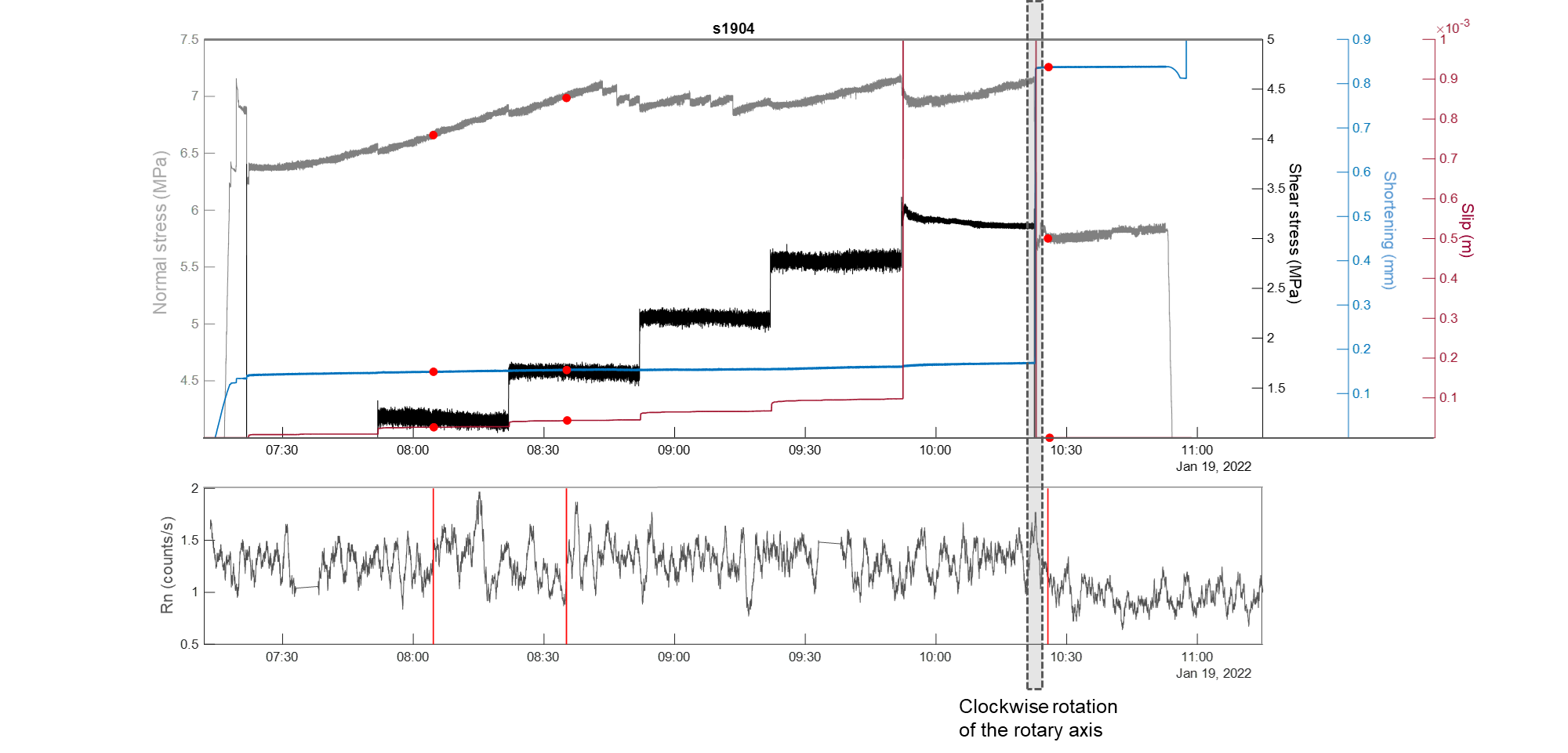


Figure S1. Paragneiss s1904. Top panel: dark grey normal stress (MPa), black shear stress (MPa), dark red slip (m), blue axial shortening (mm); red points change points (std). Bottom panel: radon, count/s moving average over 1 min, red lines change points (std).

**Figure S2:**


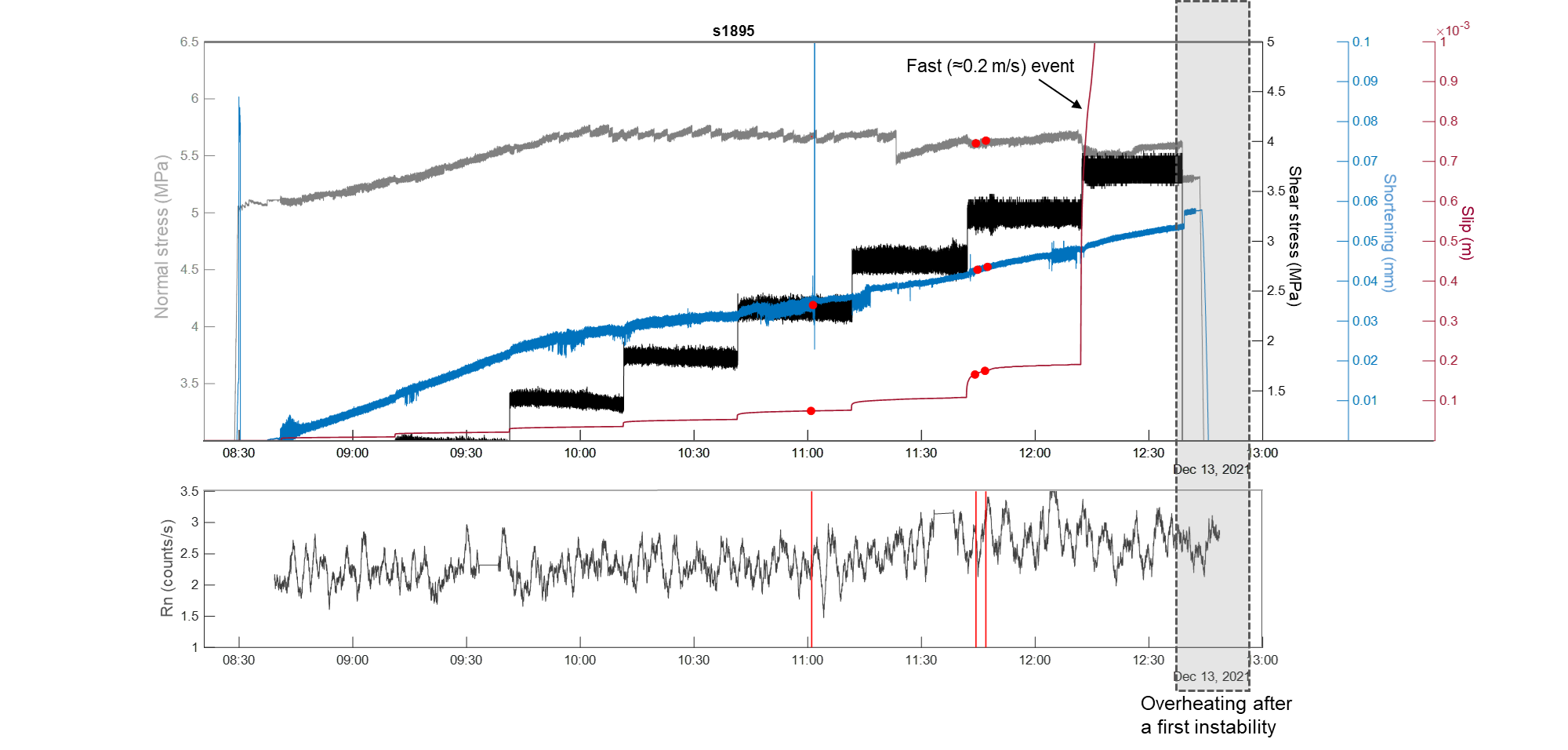


Figure S2. Granite s1895. Top panel: dark grey normal stress (MPa), black shear stress (MPa), dark red slip (m), blue axial shortening (mm); red points change points (mean). Bottom panel: radon, count/s moving average over 1 min, red lines change points (mean).

**Figure S3:**


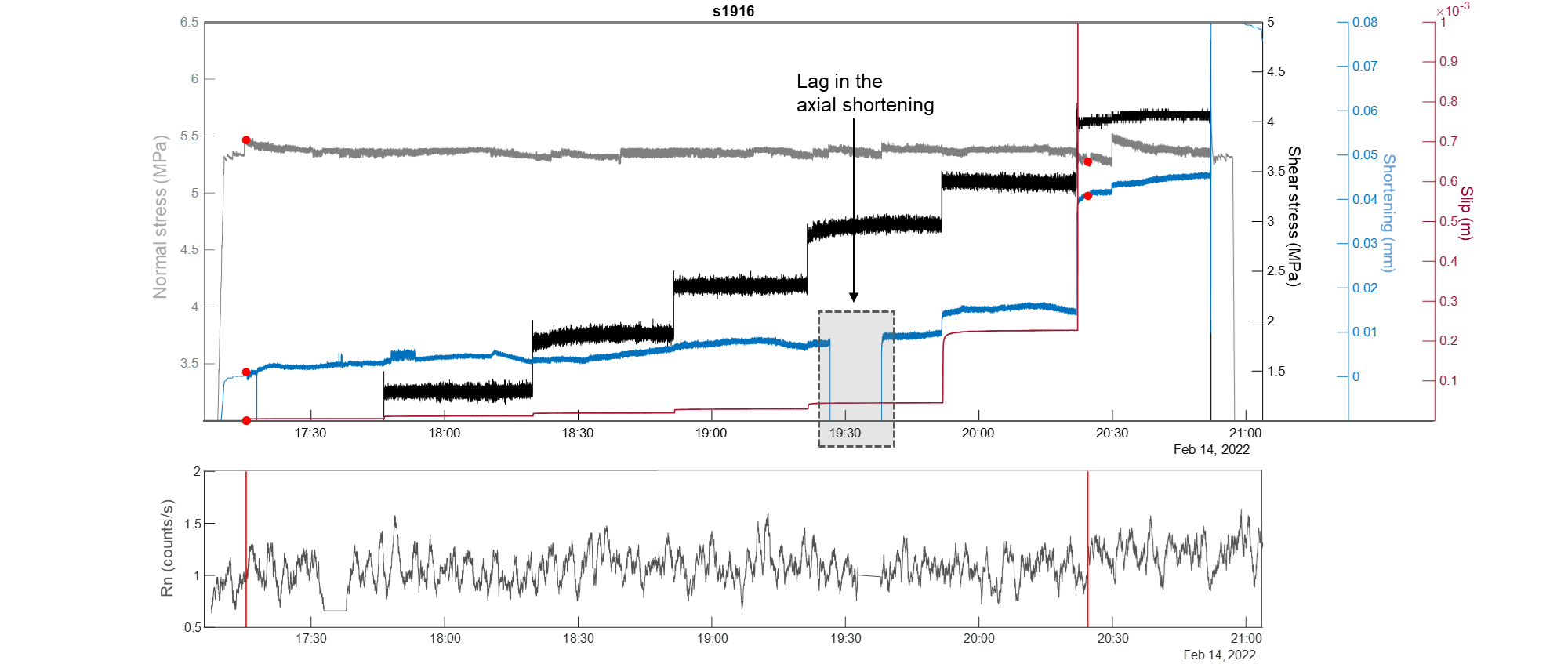


Figure S3. Orthogneiss s1916. Top panel: dark grey normal stress (MPa), black shear stress (MPa), dark red slip (m), blue axial shortening (mm); red points change points (mean). Bottom panel: radon, count/s moving average over 1 min, red lines change points (mean).

**Figure S4:**


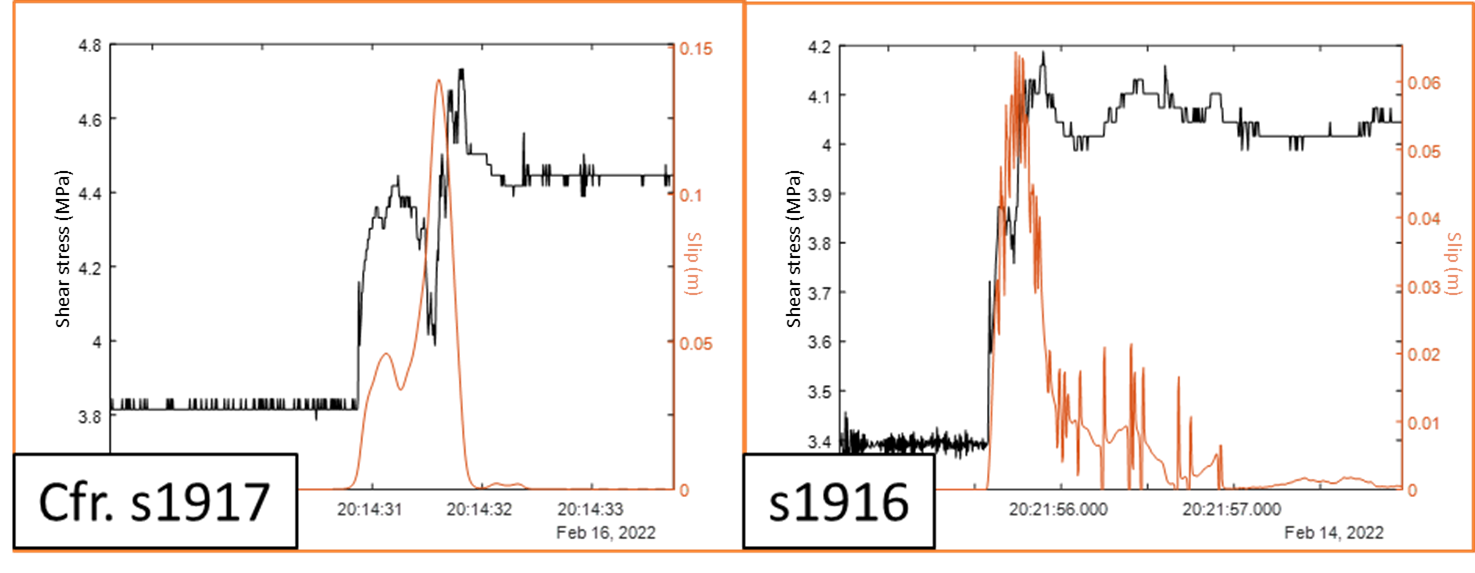


Figure S4. Shear stress (black line, MPa), slip (orange line, m). Short and fast slip events recorded before the main instability event in experiments s1917 and s1916, compared. These events reached slip velocities of 0.15 and 0.06 m/s respectively. Other events were detected by the change points analysis.

**Figure S5:**


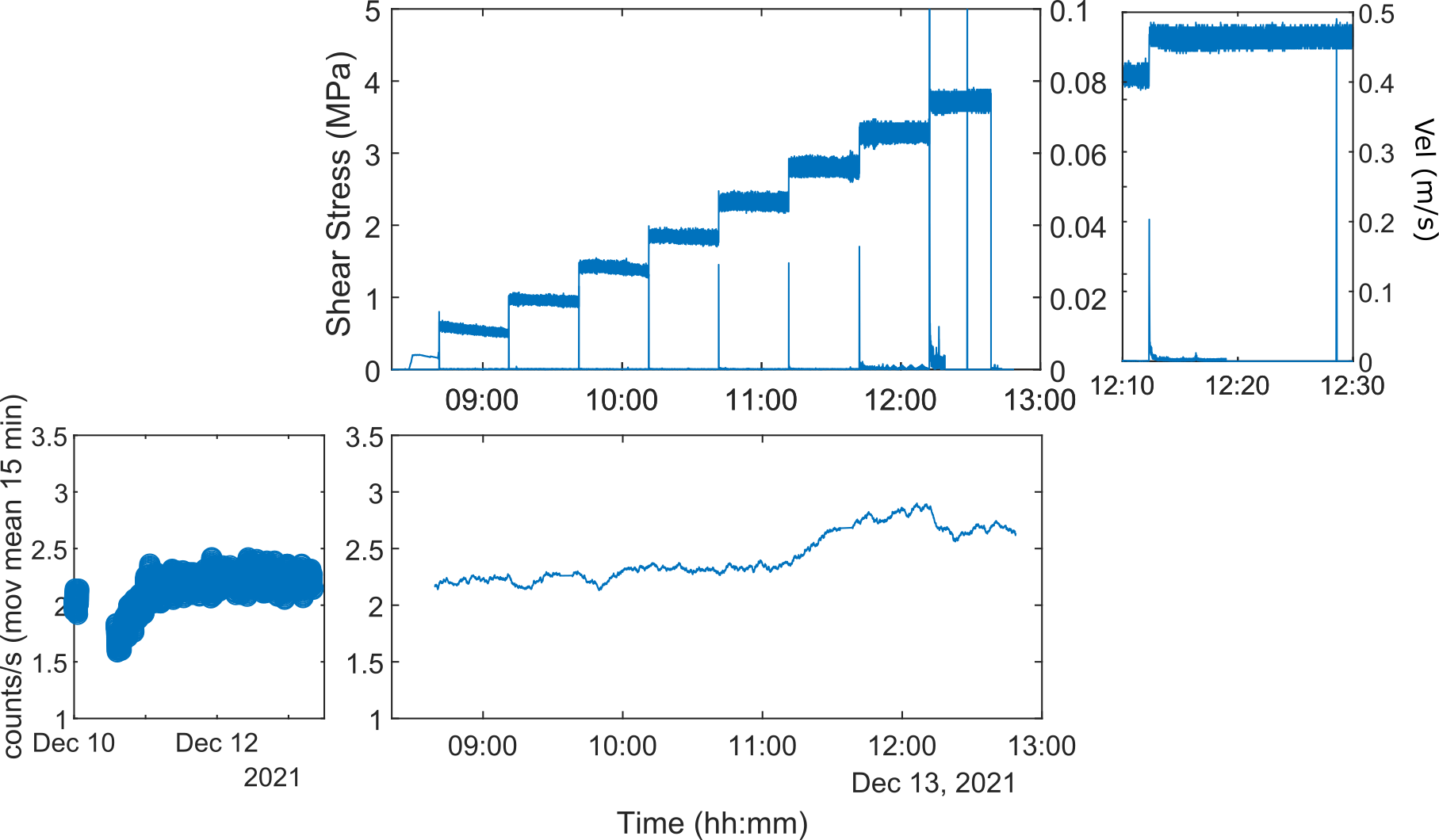


Figure S5. Top left panel: shear stress (MPa, blue) vs. time (hh:mm) for the duration of the experiment. Top right panel: a zoom of the shear stress representing one of the fast slip events recorded before the main frictional instability. Bottom left panel: the radon counts/s moving average over 15 min in the pre-experiment phase (lasted 36 hours) during and after achievement of the secular equilibrium between ^222^Rn and its short-lived progeny. Bottom right panel: the variation in radon counts/s during the experiment (lasted ca. 4 hours) with respect to the radon initial conditions.

**Figure S6:**

**
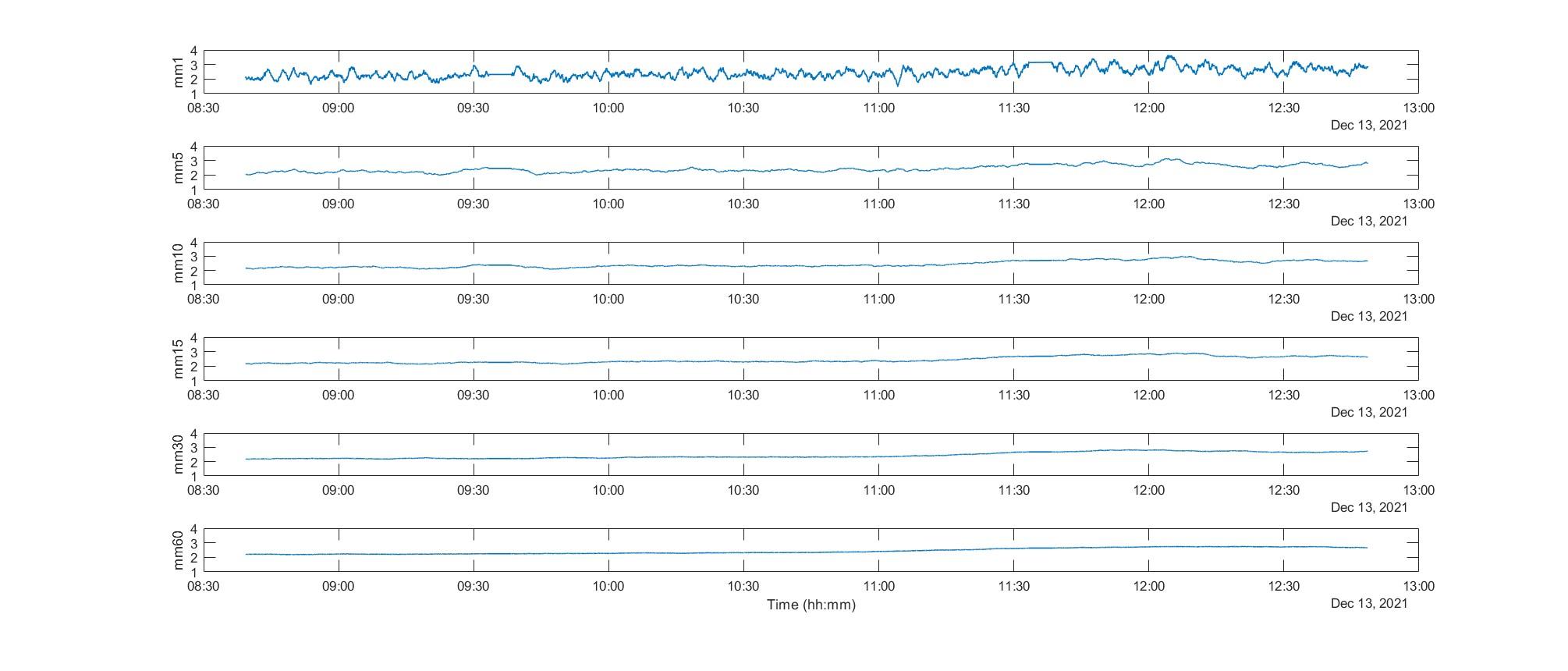
**Figure S6. One example of the different sampling rates for a granite, experiment s1895. Blue line: radon, count/s moving average over 1 min, 5 min, 10 min, 15 min, 30 min and 60 min. The change point analysis was performed using a1 min moving average.

**Figure S7:**


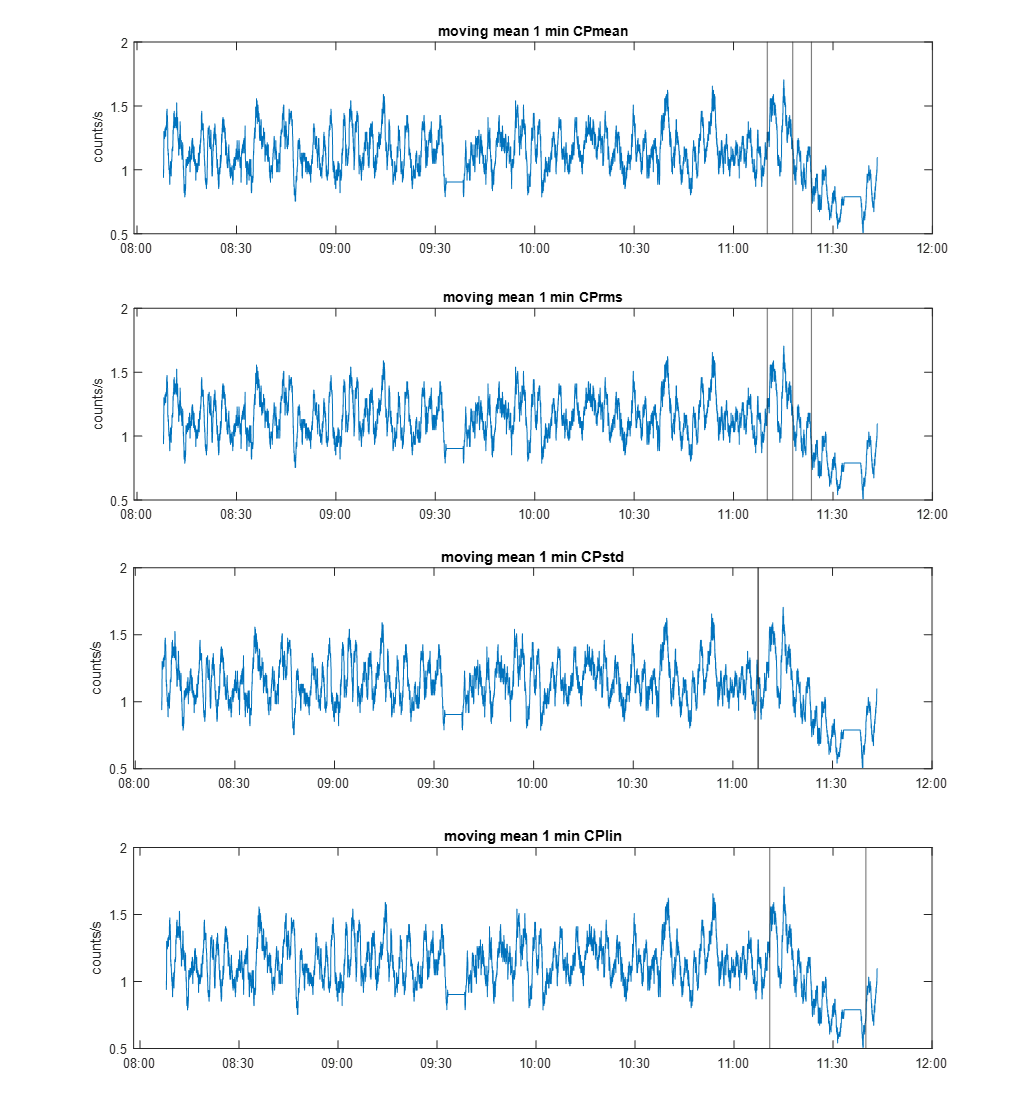


Figure S7. Paragneiss s1896. Blue line: radon, count/s moving average over 1 min, grey lines change points.

**Figure S8:**


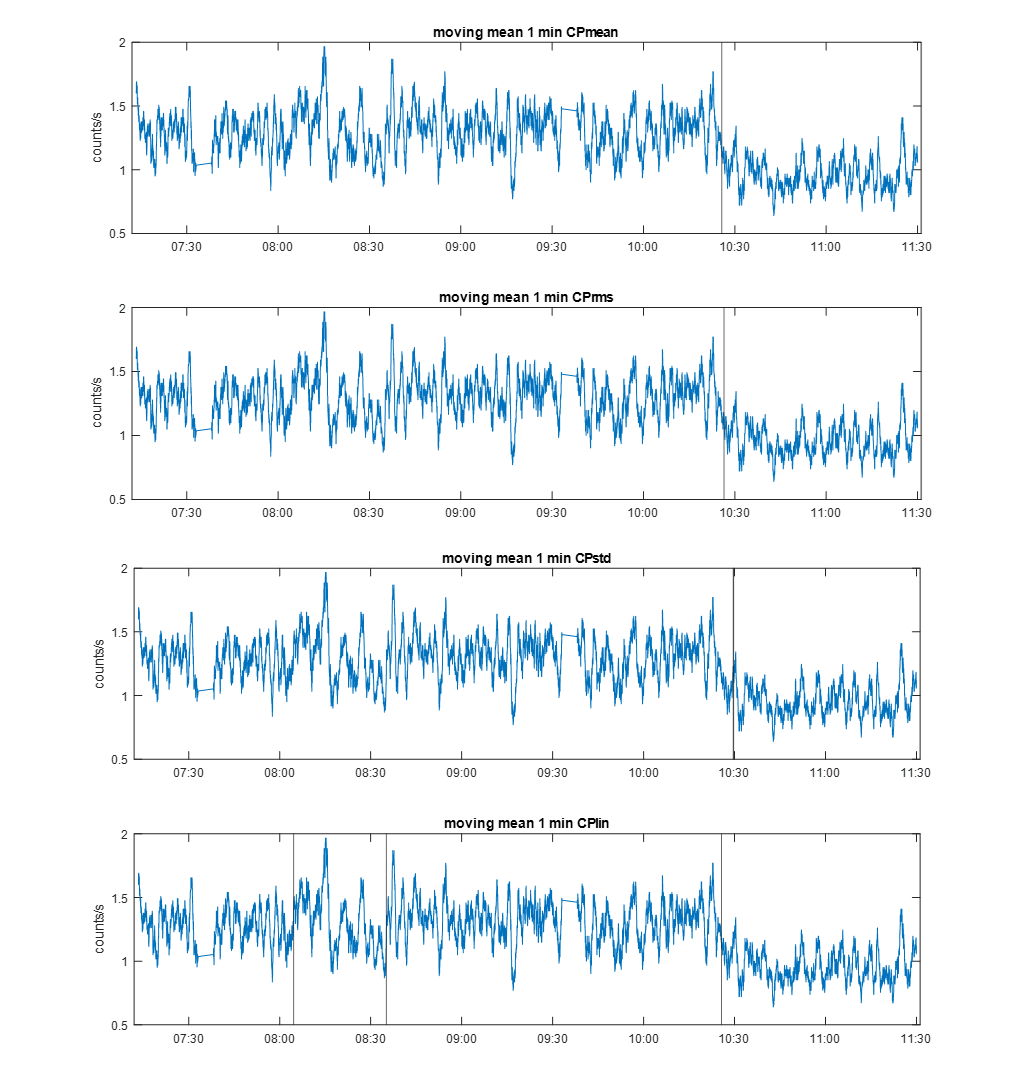


Figure S8. Paragneiss s1904. Blue line: radon, count/s moving average over 1 min, grey lines change points.

**Figure S9:**


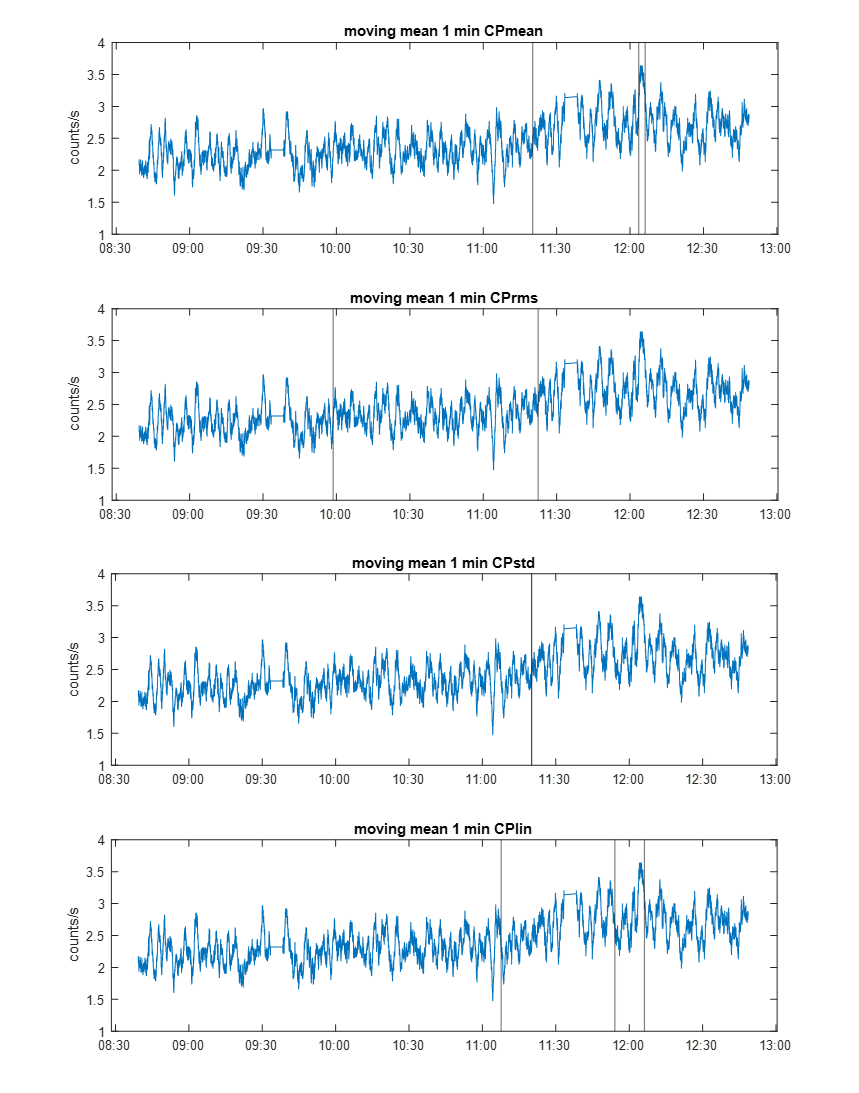


Figure S9. granite s1895. Blue line: radon, count/s moving average over 1 min, grey lines change points.

**Figure S10:**

**
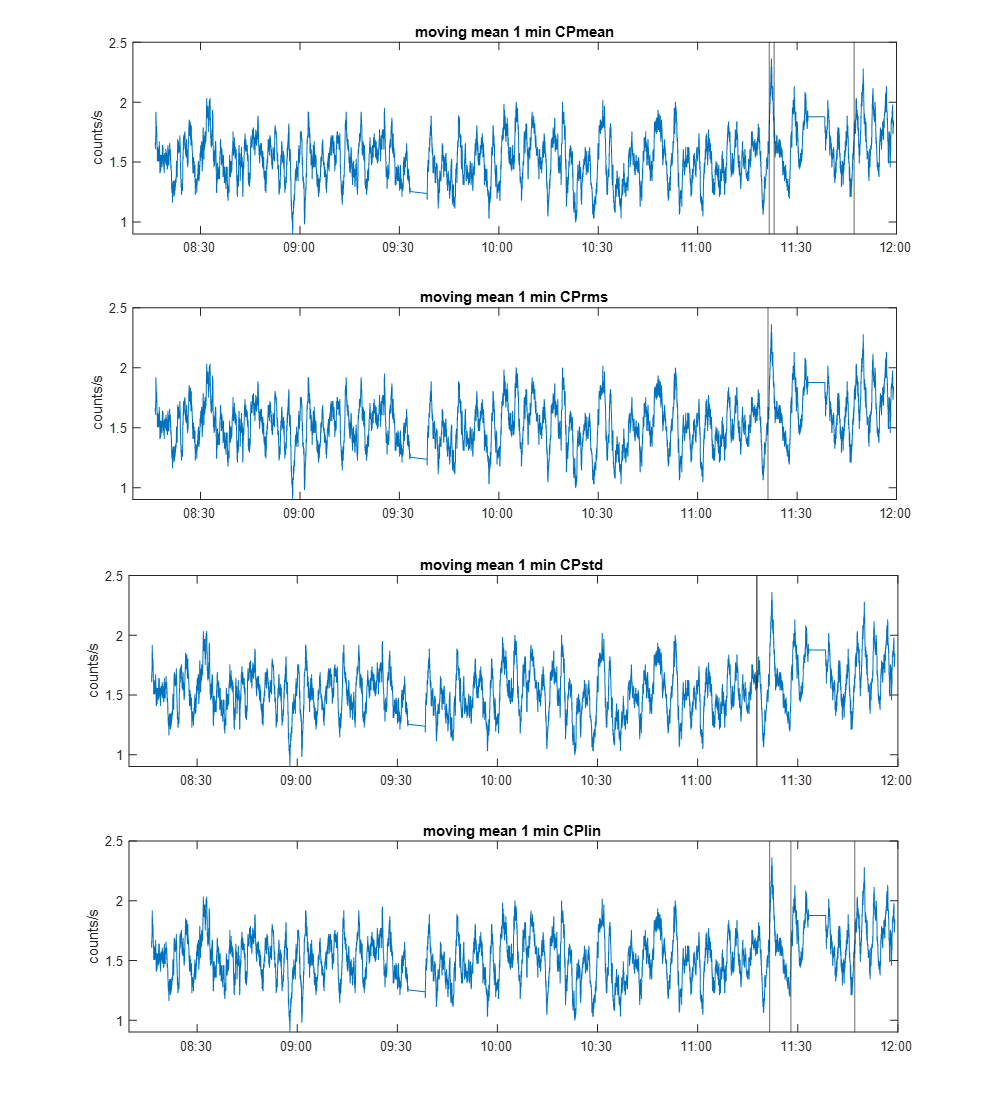
**

Figure S10. Granite s1897. Blue line: radon, count/s moving average over 1 min, grey lines change points.

**Figure S11:**


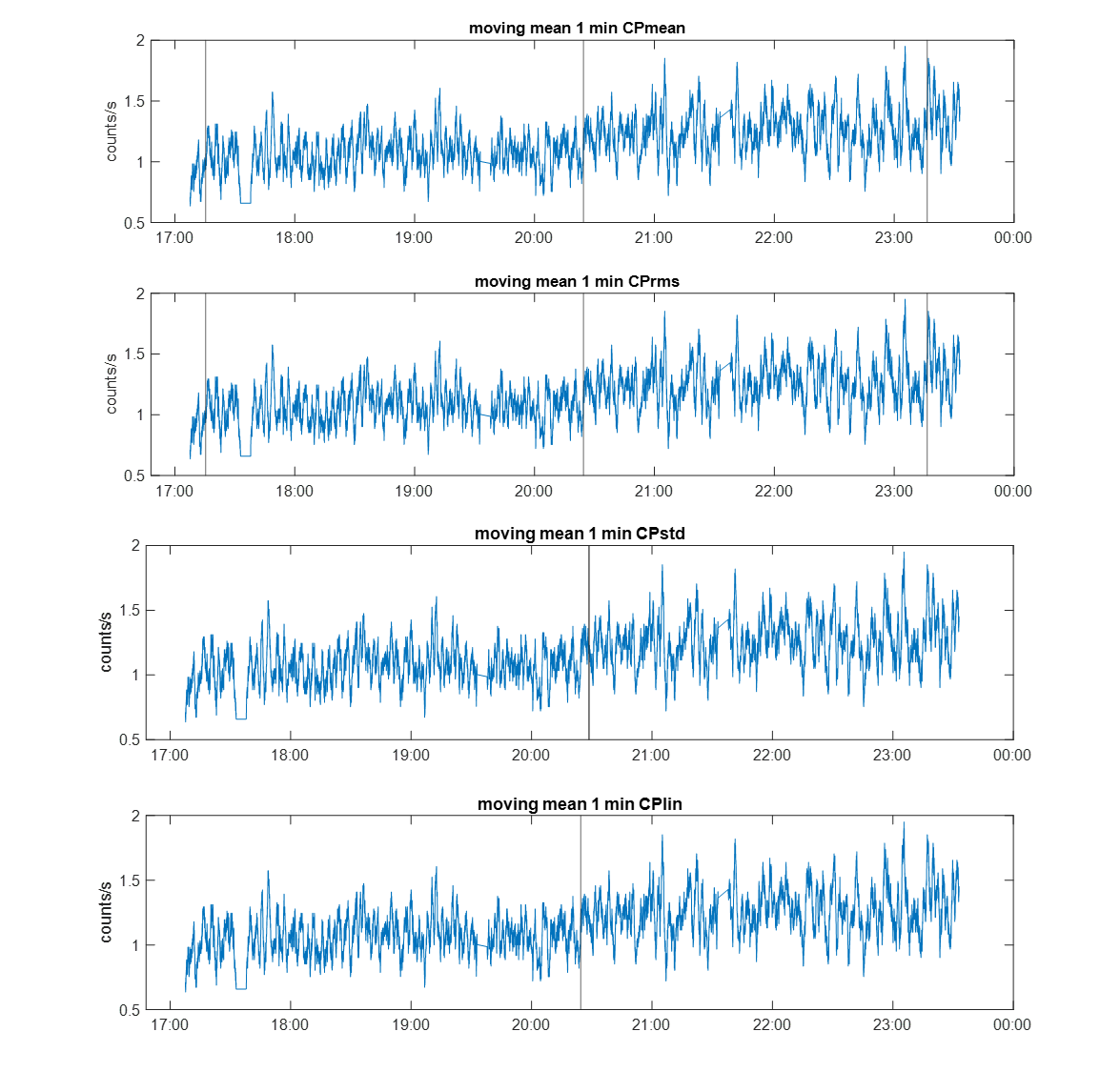


Figure S11. Orthogneiss s1916. Blue line: radon, count/s moving average over 1 min, grey lines change points.

**Figure S12:**

**
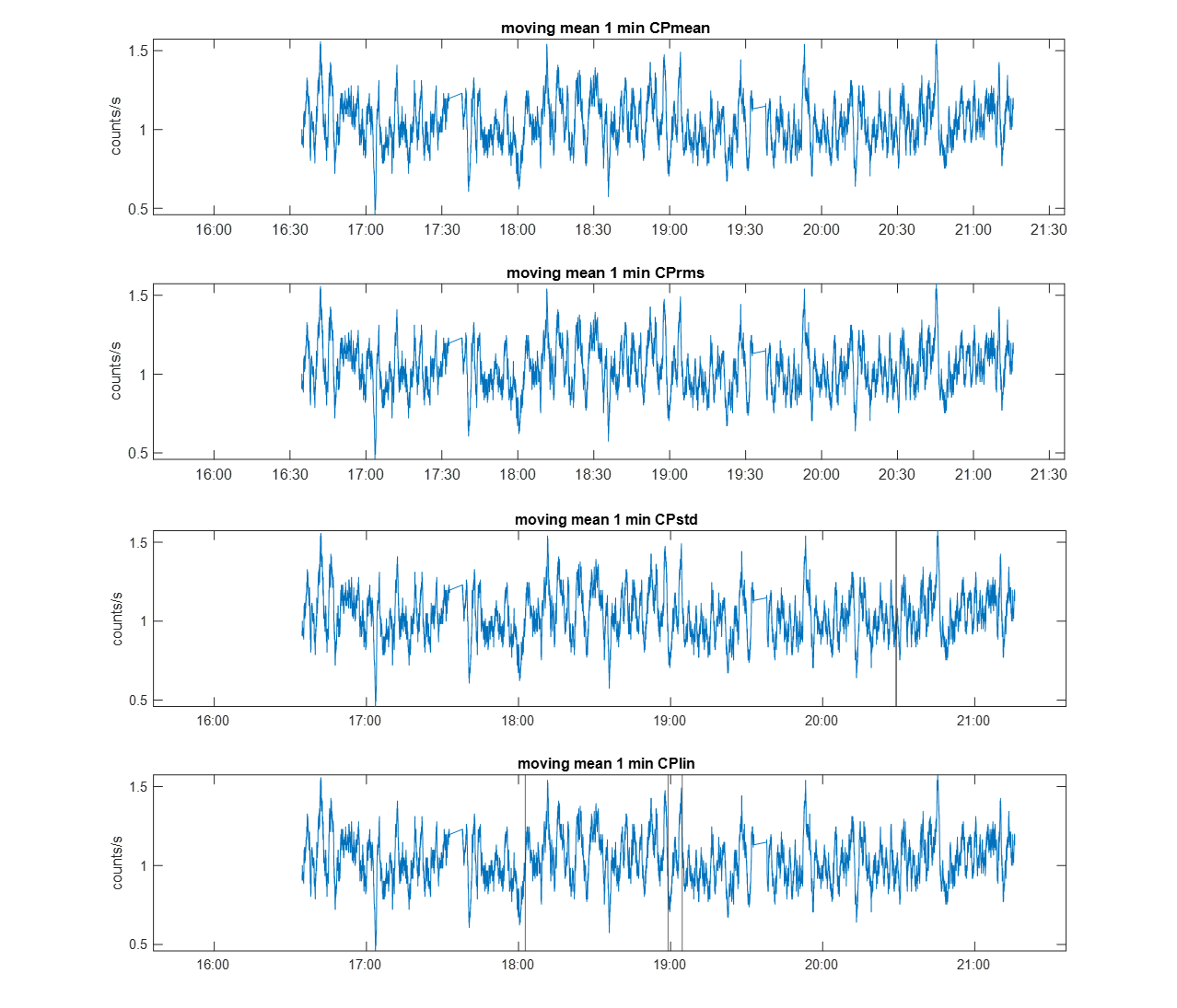
**

Figure S12. Orthogneiss s1917. Blue line: radon, count/s moving average over 1 min, grey lines change points.
